# Autocrine glutamatergic transmission for the regulation of embryonal carcinoma stem cells

**DOI:** 10.18632/oncotarget.9973

**Published:** 2016-06-13

**Authors:** Lin Teng, Hui-Min Lei, Fan Sun, Shi-Min An, Ya-Bin Tang, Shuang Meng, Cong-Hui Wang, Ying Shen, Hong-Zhuan Chen, Liang Zhu

**Affiliations:** ^1^ Department of Pharmacology and Chemical Biology, Basic Medicine Faculty of Shanghai Jiao Tong University School of Medicine, Shanghai 200025, China; ^2^ Shanghai Universities Collaborative Innovation Center for Translational Medicine, Shanghai 200025, China; ^3^ Department of Pharmacy, Renji Hospital Affiliated to Shanghai Jiao Tong University School of Medicine, Shanghai 200127, China; ^4^ Present address: Department of Cardiology, The First College of Clinical Medical Sciences, China Three Gorges University, Hubei 443003, China

**Keywords:** autocrine, glutamatergic, signaling, embryonal carcinoma stem cell, transmission

## Abstract

Glutamate behaves as the principal excitatory neurotransmitter in the vertebrate central nervous system and recently demonstrates intercellular signaling activities in periphery cancer cells. How the glutamatergic transmission is organized and operated in cancer stem cells remains undefined. We have identified a glutamatergic transmission circuit in embryonal carcinoma stem cells. The circuit is organized and operated in an autocrine mechanism and suppresses the cell proliferation and motility. Biological analyses determined a repertoire of glutamatergic transmission components, glutaminase, vesicular glutamate transporter, glutamate NMDA receptor, and cell membrane excitatory amino-acid transporter, for glutamate biosynthesis, package for secretion, reaction, and reuptake in mouse and human embryonal carcinoma stem cells. The glutamatergic components were also identified in mouse transplanted teratocarcinoma and in human primary teratocarcinoma tissues. Released glutamate acting as the signal was directly quantified by liquid chromatography coupled with tandem mass spectrometry (LC-MS/MS). Genetic and pharmacological abolishment of the endogenously released glutamate-induced tonic activation of the NMDA receptors increased the cell proliferation and motility. The finding suggests that embryonal carcinoma stem cells can be actively regulated by establishing a glutamatergic autocrine/paracrine niche via releasing and responding to the transmitter.

## INTRODUCTION

Glutamate is the principal excitatory transmitter in the vertebrate central nervous system. Glutamatergic neurons synthesize glutamate mainly from glutamine by glutaminase (GLS), then loading it into presynaptic vesicles via vesicular glutamate transporter (VGLUT) for its secretion. The released glutamate binds to and activates its cognate receptors (glutamate receptors, GluRs), the ionotropic glutamate receptor (iGluR) subtypes AMPA (a-amino-3-hydroxy-5-methyl-4-isoaxazolepropionate acid), Kainat, NMDA (N-methyl-D-aspartate) and Delta receptors [1], and the metabotropic glutamate receptor (mGluR) subtypes [2]. The cell membrane excitatory amino-acid transporter (EAAT) then takes the released glutamate up into astrocytes and neurons, terminating the glutamatergic signal.

In addition to its action on synaptic transmission and neurogenesis, outside the central nervous system, non-neuronal glutamatergic transmission has been discovered [3–6]. Malignant cells, such as those in melanoma, colorectal carcinoma, hepatocellular carcinoma, and prostate carcinoma are modulated by the transmission system where glutamate acts as an intercellular signaling factor [7–10]. However, knowledge about the role of glutamatergic signaling in cancer development and progression is still in its infancy [11, 12] and how the glutamatergic transmission circuit is organized and operated in cancer stem cells remains undefined.

Here, we have identified that embryonal carcinoma stem (ECS) cells, the cancer stem cells of teratocarcinoma [13–15], possess an internal glutamatergic transmission circuit. The circuit is organized and operated in an autocrine mechanism and suppresses the cancer stem cell population and motility.

## RESULTS

### Embryonal carcinoma stem cells express glutamatergic transmission output and reuptake components

**Figure 1:**
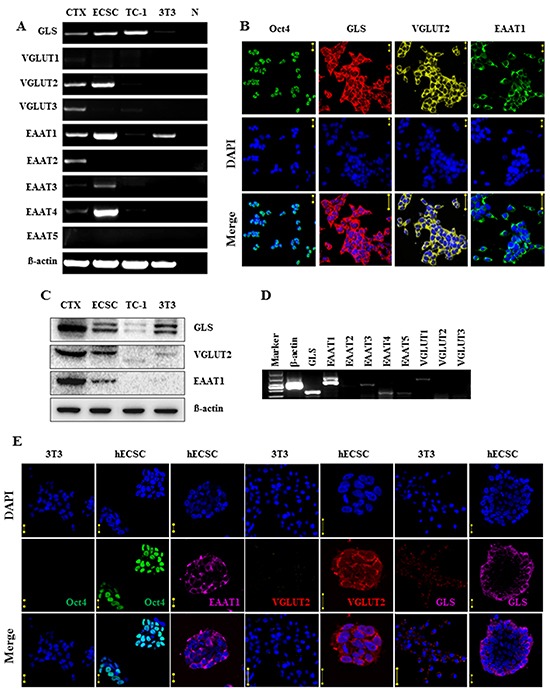
Expression of glutamatergic transmission output and reuptake components in embryonal carcinoma stem cells **A.** RT-PCR analysis of glutamate synthesis enzyme GLS, vesicular transporters VGLUT1-VGLUT3, cell membrane transporters EAAT1-EAAT5 of mouse ECS cells. CTX, cerebral cortex tissue control. ECSC, embryonal carcinoma stem cell, TC-1, lung cancer cell control. 3T3, NIH/3T3 cell control. N, cDNA free control. **B.** Immunofluorescence staining analysis of Oct4, GLS, VGLUT2, and EAAT1 of mouse ECS cells. DAPI represents cell nucleus position; Oct4 is a pluripotent marker. Scale bar: 20 μm. **C.** Western blot analysis of GLS, VGLUT2, and EAAT1 of mouse ECS cells. **D.** RT-PCR analysis of glutamatergic components in human ECS cells. **E.** Immunofluorescence staining analysis of glutamatergic components in human ECS cells. DAPI represents cell nucleus position; Oct4 is a pluripotent marker. Scale bar: 20 μm. NIH/3T3 as control cells. ECSC, embryonal carcinoma stem cell; hECSC, human embryonal carcinoma stem cell.

RT-PCR analysis revealed that mouse ECS cells expressed the transcripts of glutamate synthesis enzymes GLS; vesicular transporter VGLUT2; and membrane transporters EAAT1, EAAT3, and EAAT4 (Figure 1A). The expression of the glutamatergic transmission components was confirmed by immunocytofluorescence staining analysis (Figure 1B) and western blot assay (Figure 1C); the GLS, VGLUT2, and EAAT1 proteins were identified (Figure 1B and 1C), with the degree of expression comparable to that in the cerebral cortex (Figure 1C). Human ECS cells also were detected to express the glutamatergic transmission components GLS, VGLUT, and EAATs in RT-PCR assay (Figure 1D) and in immunocytofluorescence staining analysis (Figure 1E). The expression levels of the signaling components were far less in NIH/3T3 cells (Figure 1C and 1E), indicating their selective expression in ECS cells.

**Figure 2:**
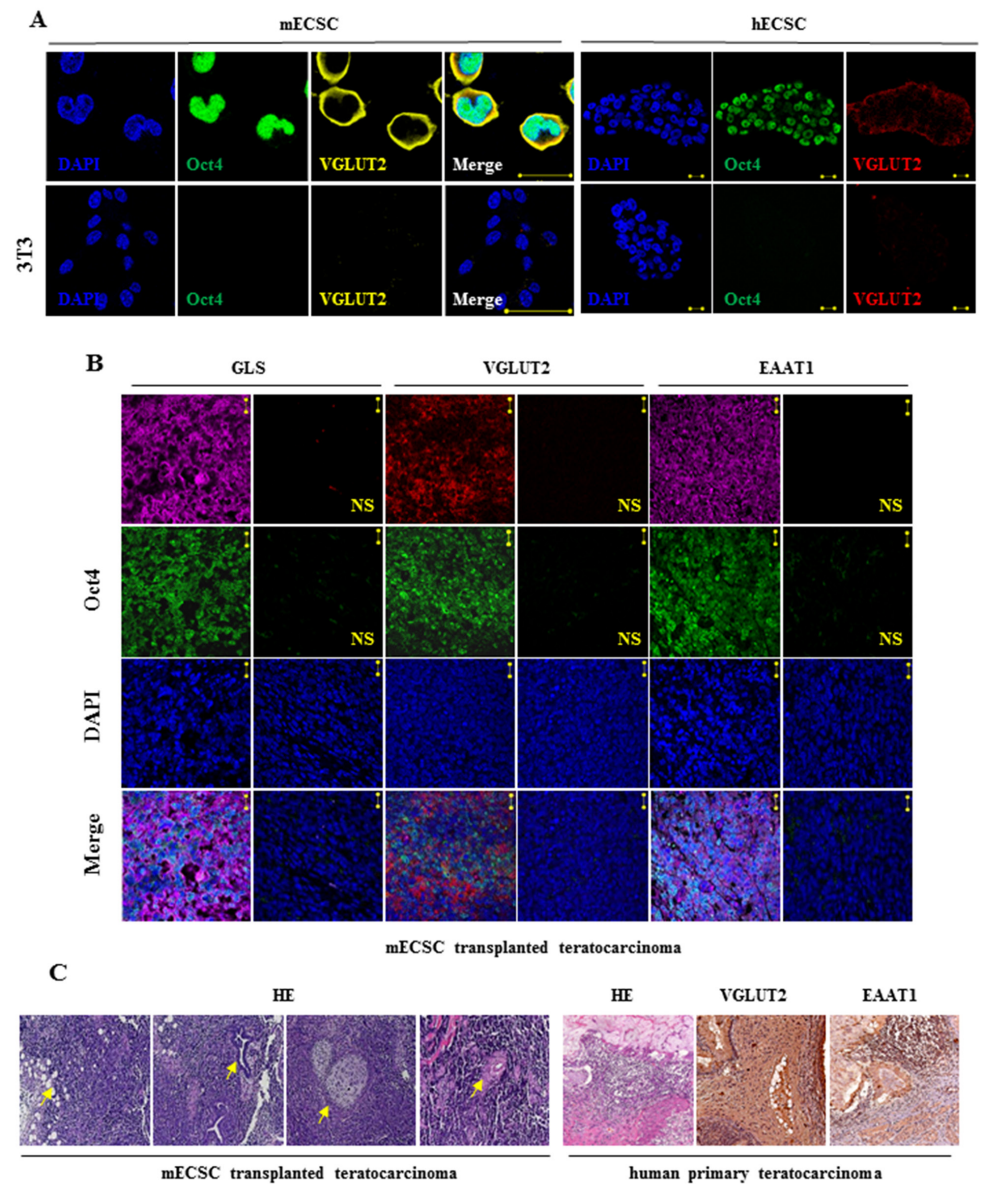
Glutamatergic markers in embryonal carcinoma stem cells and in teratocarcinoma **A.** Colocalization of glutamatergic marker VGLUT and pluripotent marker Oct4 in the same ECS cells detected by immunofluorescence staining confocal imaging assay. DAPI represents cell nucleus position. NIH/3T3 as control cells. Scale bar: 20 μm. **B.** Immunofluorescence staining analysis of Oct4, GLS, VGLUT2, and EAAT1 in mouse ECS cell-transplanted teratocarcinoma. DAPI represents cell nucleus position; Oct4 is a pluripotent marker. NS, first antibody free non-specific control. Scale bar: 20 μm. **C.** Hematoxylin and eosin (HE) staining of mouse ECSC transplanted teratocarcinoma tissue (left panel; the arrow from the left image to the right indicates adipose, neural tube, bone and cartilage, and epithelium, respectively; with 10 x magnification objective lens) and HE staining and immunohistochemical (IH) staining for the glutamatergic markers in human primary teratocarcinoma tissue (right panel; with 20 x magnification objective lens; the vesicular transporter VGLUT is a glutamatergic markers and the cell membrane transporter EAAT is a glutamatergic transmission reuptake component).

The glutamatergic marker VGLUT colocalized with the pluripotent marker Oct4 in a same ESC cell (Figure 2A). The components were also identified in ECS cells in mouse transplanted teratocarcinoma tissue (Figure 2B), and in human primary teratocarcinoma tissue (Figure 2C, right panel).

### Embryonal carcinoma stem cells express glutamatergic transmission input components

**Figure 3:**
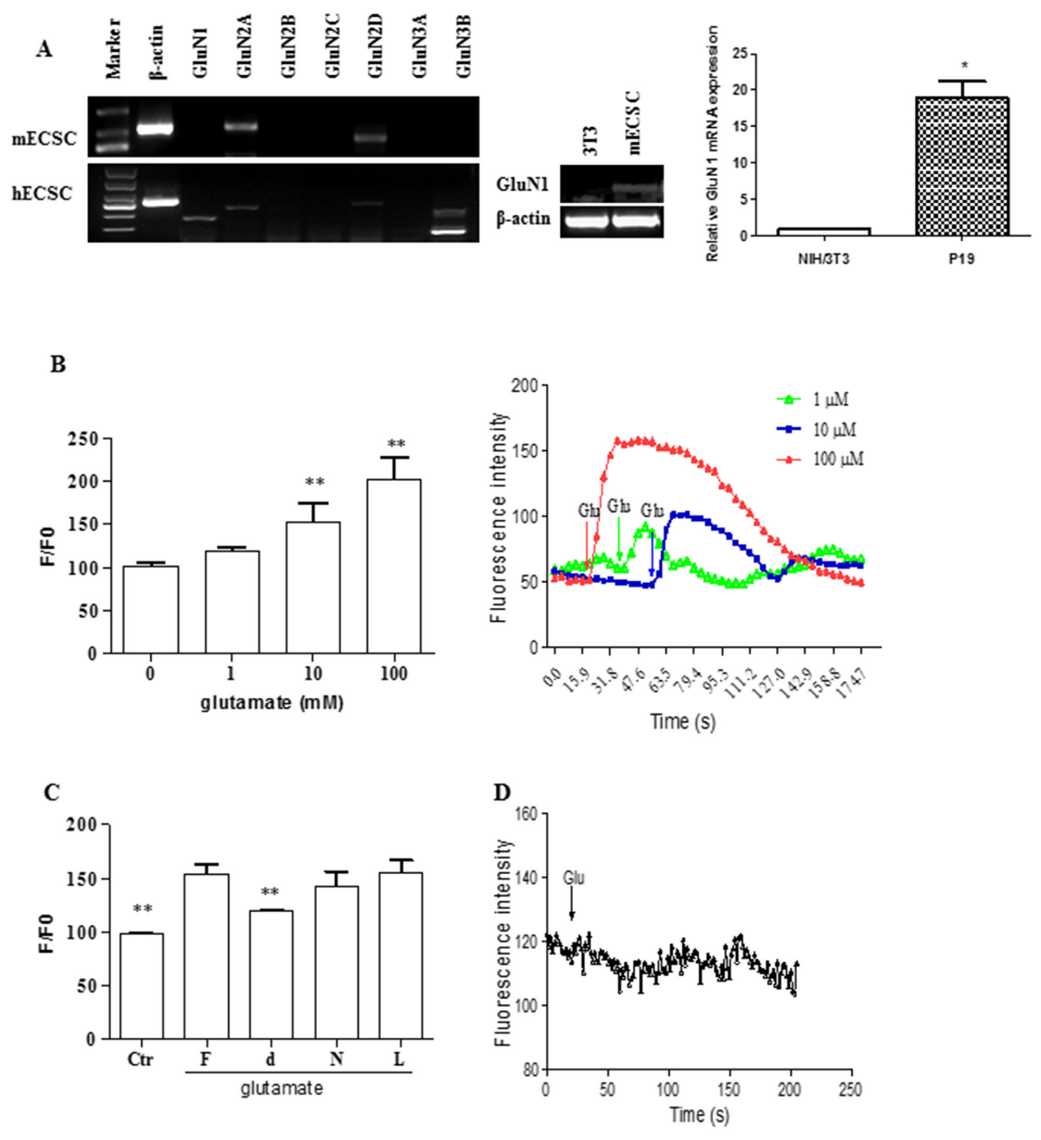
Functional NMDA receptors in embryonal carcinoma stem cells **A.** RT-PCR and/or RT-qPCR analysis of glutamatergic NMDA receptor subunits in ECS cells. The band for mECS cell GluN1 subunit in left panel is too faint but clearly visible and detected when the subunit transcript was particularly assayed by RT-PCR (middle panel) and RT-qPCR (right panel) analysis. NIH/3T3 as control cells. *, p < 0.05, compared with NIH/3T3 group. **B.** Glutamate-induced increase of [Ca^2+^]i in mouse ECS cells. Data represent mean ± s.e.m from at least three independent experiments. **, p < 0.01, compared with non-glutamate administrated ECS cell group. Graph in the right panel shows the representative responses of the glutamate-induced increase of [Ca^2+^]i. **C.** Dependence of NMDA receptors of the effect of glutamate-induced increase of [Ca^2+^]i in mouse ECS cells. F, antagonist free; d, d-AP5, a NMDA receptor selective antagonist; N, NBQX, an AMPA/Kainate receptor selective antagonist; L, LY341495, a mGluR antagonist. The cells were pretreated with 1 μM each of the antagonists for 15 min before the glutamate introduction. Data represent mean ± s.e.m from at least three independent experiments. **, p < 0.01 compared with corresponding antagonist-free control cells. **D.** Non-induction of the increase of [Ca^2+^]i in NIH/3T3 cells.

The transcripts of the receptor subunits GluN1, GluN2A and GluN2D for NMDA type of ionotropic glutamate receptors (iGluRs) were identified in mouse ECS cells (Figure 3A). Human ECS cells expressed GluN1, GluN2A, GluN2D, and GluN3B transcripts (Figure 3A). Functional NMDA receptors are calcium permeable channels. We measured the cytosolic free calcium change to determine whether the receptors are capable of responding to their cognate agonist. Glutamate at concentrations above 1 μM induced an increase of [Ca^2+^]i in a dose-dependent manner in ECS cells (Figure 3B). The effect was selectively blocked by the preincubation of NMDA receptor antagonist d-AP5 (Figure 3C), indicating the NMDA receptor-depended profile. In contrast, NIH/3T3 cells did not respond to glutamate (Figure 3D).

### Embryonal carcinoma stem cells release glutamate

**Figure 4:**
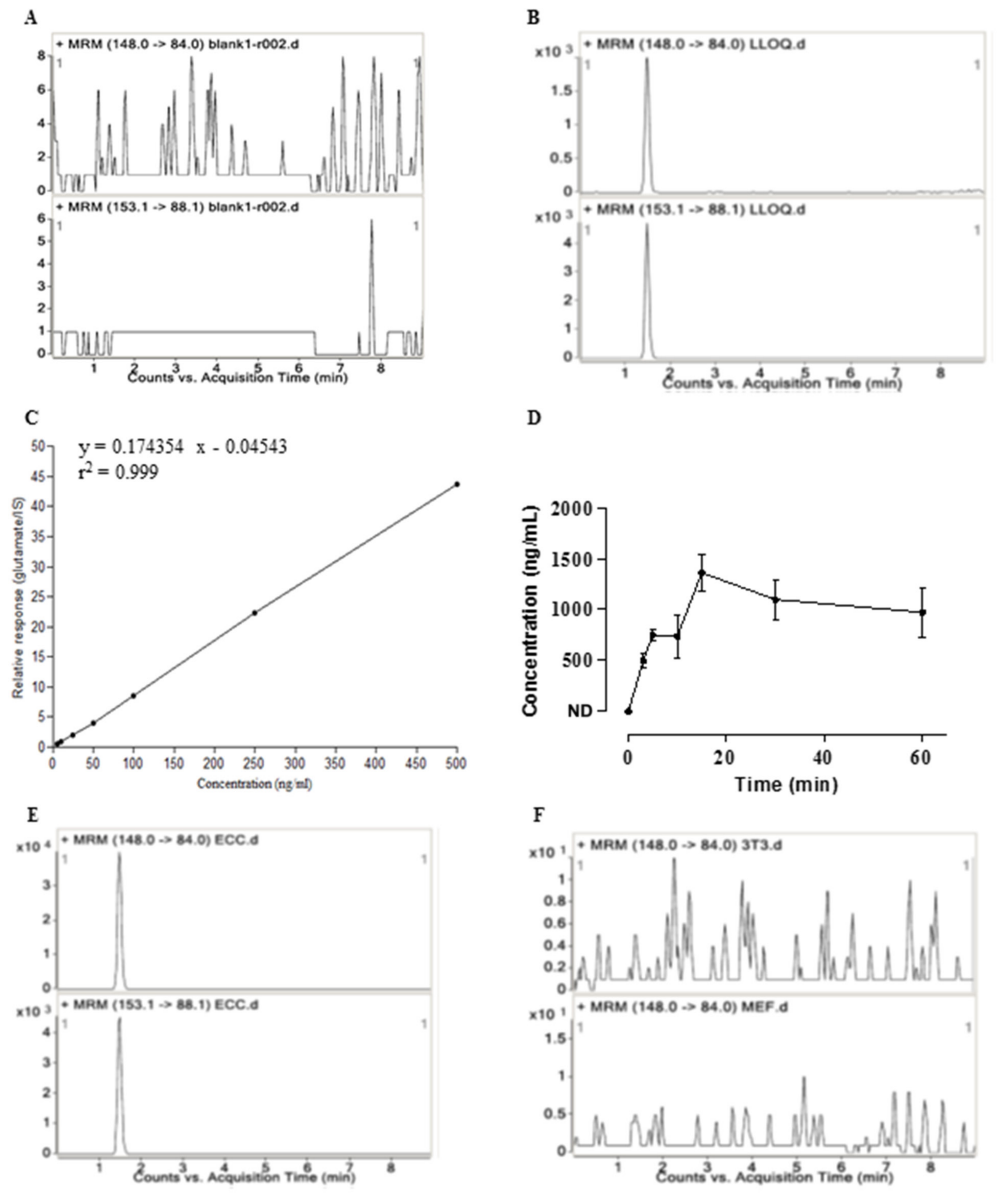
Release of glutamate by embryonal carcinoma cells determined by HPLC-MS/MS **A.** Representative MRM chromatograms for glutamate (up panel) and IS (down panel) of a blank KHB sample (non-spiked). **B.** Representative MRM chromatograms for glutamate (up panel) and IS (down panel) spiked with glutamate at lower limit of quantitation (LLOQ) level. The chromatograms showed no interfering compound at the retention time of glutamate and IS. **C.** Typical daily calibration curve for glutamate describes a good linear relationship between the peak area ratios of analyte to IS (y) and analyte level (x). **D.** Release of glutamate from ECS cells. Concentrations are expressed as mean ± s.e.m for ECS cells (n=2 in duplicate samples). ND, not detected. **E.** Representative multiple reaction monitoring (MRM) chromatograms for glutamate (up panel) and IS (down panel) in the supernatants of ECS cells. **F.** Representative MRM chromatograms for glutamate in the supernatants of NIH/3T3 cells (up panel) and MEFs (down panel) as the control cells.

An analytic chemistry approach based on HPLC-MS/MS was established to quantify the glutamate released from ECS cells into the intercellular milieu. The method was validated in terms of selectivity and linearity. The chromatogram of the blank matrix showed no interfering compound for glutamate and IS in the matrix (Figure 4A), whereas the signal-to-noise (S/N, peak to peak) of the analytes in the glutamate and IS spiked sample was more than 50 at the lower limit of quantification (LLOQ) (Figure 4B). Therefore, the selectivity of the approach was well acceptable. The calibration curve showed a good linear correlation over the range of 5-500 ng/mL (y = 0.174354x - 0.04543, r^2^ = 0.999) (Figure 4C).

Glutamate was determined to be released by ECS cells (Figure 4D and 4E). The concentration in the intercellular milieu was 496.31 ng/mL at 3 min, increasing to 750.93 ± 59.73 ng/mL at 5 min and 1360.59 ± 180.88 ng/mL at 15 min and kept the similar concentrations until 60 min (Figure 4D). The release of glutamate depended on calcium because the release was abolished when the ECS cells were buffered in Ca^2+^-free KHB solution (data not shown). In contrast to ECS cells, the level of glutamate released from NIH/3T3 cells (Figure 4F, up panel) or MEFs (Figure 4F, down panel) was below the detection limit.

### Autocrine glutamate suppresses embryonal carcinoma cell proliferation and migration

Embryonal ECS cells express glutamatergic transmission output and input components and release glutamate signal, establishing an autocrine circuit. If the abolishment of the receptor activation, such as by genetic knockdown or pharmacological blockade of the receptors without concomitant exogenous administration of the agonists, can change the cell functional phenotypes, the autocrine mechanism of the glutamatergic transmission will be further confirmed.

**Figure 5:**
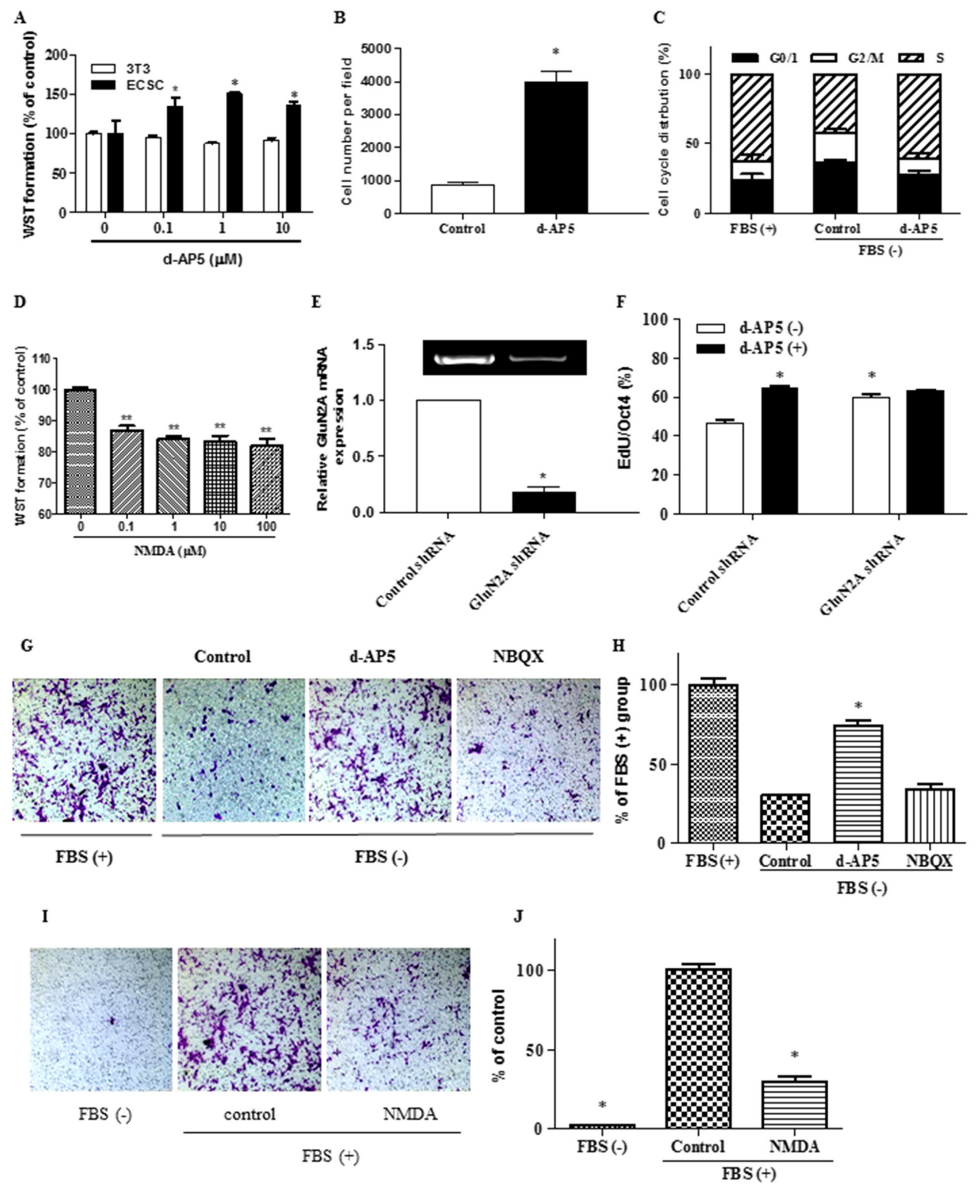
Regulation of embryonal carcinoma stem cell proliferation and migration by endogenous glutamate signaling **A.** Increase of ECS cell proliferation by the inhibition of NMDA receptors tested by WST assay. The cells were treated for 24 h. Viability is given as a percentage of the control value. *, p < 0.05 compared with non-treatment control. 3T3, NIH/3T3 control cells. **B.** Increase of ECS cell proliferation by the inhibition of NMDA receptors assayed by cell count analysis per field. The cells were treated with 1 μM d-AP5 for 24 h. **C.** Increase of the proportion of ECS cells in S phase and decrease of those in G0/1 and G2/M phase by the inhibition of NMDA receptors assayed by flow cytometric cell-cycle analysis. The cells were treated with 1 μM d-AP5 for 24 h. **D.** Decrease of ECS cell proliferation by the activation of NMDA receptors tested by WST assay. The cells were cultured in medium containing 10% FBS. The cells were treated with NMDA for 48 h. **E.** RT-PCR (gel blot image) and qPCR (bar graph) analysis of the knockdown of glutamate receptor subunit GluN2A. **F.** Effects of NMDA receptor knockdown on background proliferation and d-AP5-induced pro-proliferation in ECS cells quantitatively assayed of EdU incorporation into DNA by High-Content Analysis (HCA). d-AP5 was used at 10 μM for 24 h. **G.** and **H.** Increase of mobility of ECS cells by the inhibition of NMDA receptors assayed by transwell migration analysis. Magnification, 200 ×. Quantifications are shown in (H). Assays were performed three times using triplicate wells. The cells were treated with 1 μM d-AP5 for 24 h. FBS, fetal bovine serum, as migration-inducing control. **I.** and **J.** Decrease of the mobility of ECS cells by the exogenous introduction of 10 μM NMDA for 24 h assayed by transwell migration analysis. Quantifications are shown in (J). Magnification, 200 ×. d-AP5, a NMDA receptor selective antagonist; NBQX, an AMPA/Kainate receptor selective antagonist. Cells were cultured in exogenous glutamate-free media unless otherwise indicated. Data represent mean ± s.e.m from at least three independent experiments. For HCA, quantification was averaged from at least 6 randomly selected microscopic fields. For WST assay, results were analyzed from 5 independent experiments performed in 6 parallel well points in 96-wells. For migration assay, quantification was averaged from at least 6 randomly selected microscopic fields.

**Figure 6:**
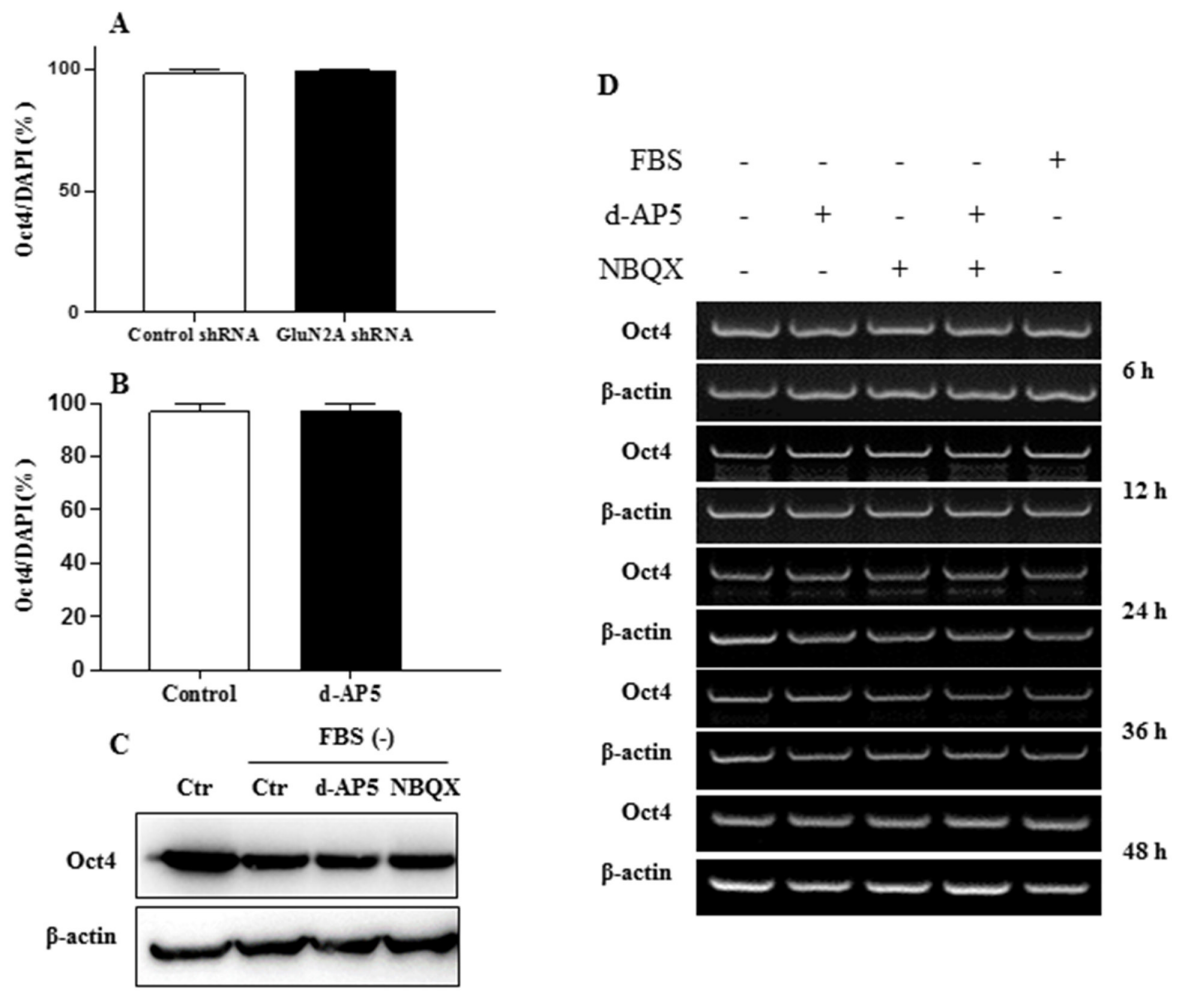
Effects of NMDA receptors on embryonal carcinoma stem cell pluripotency **A.** Effects of the knockdown of NMDA receptor GluN2A subunit on the expression of pluripotent marker Oct4 of the ECS cells quantitatively assayed by HCA. **B.** Expression of Oct4 after the inhibition of NMDA receptors of ECS cells detected by HCA. The cells were treated with 10 μM antagonist for 24 h. **C.** Expression of Oct4 after the inhibition of NMDA and AMPA receptors of ECS cells detected by Western blot. The cells were treated with 1 μM each antagonist for 24 h. **D.** Time kinetics of pluripotent marker Oct4 expression after the inhibition of NMDA and AMPA receptors of ECS cells detected by RT-PCR. The concentration of each antagonist is 1 μM. Data represent mean ± s.e.m from at least three independent experiments. For HCA quantification was averaged from at least 6 randomly selected microscopic fields.

Application of glutamatergic NMDA receptor antagonist d-AP5 selectively led to a concentration-dependent increase of the cell number assayed by WST and cell counting analysis (Figure 5A and 5B). Flow cytometric cell-cycle assay indicated that the blocking of NMDA receptors raised the proportion of cells in S phase while decreased those in G0/1 and G2/M phase (Figure 5C). In line with this, exogenous administration of NMDA inhibited cell proliferation (Figure 5D). We further used RNA interference to knock down the expression of GluN2A (Figure 5E), the orthosteric subunit in the NMDA receptor for glutamate ligand binding and subsequent functional initiation [25,26]. The genetic ablation of GluN2A in ECS cells significantly increased DNA synthesis (Figure 5F) without alteration of Oct4 expression level (Figure 6A), as determined by High-Content Analysis (HCA) of EdU incorporation into DNA and Oct4 protein expression, respectively. Consistent with the genetic modulation assay, pharmacological disruption of tonic NMDA or AMPA/Kainate receptor activation did not alter the levels of the pluripotent markers Oct4 in ECS cells as shown in HCA, western blot and RT-PCR assays (Figure 6B, 6C, and 6D). In GluN2A ablated cells, the pro-proliferative effect of d-AP5 was abrogated (Figure 5F), confirming that the effect was dependent on NMDA receptors. In addition to the modulation of cell proliferation, NMDA receptor blocking took effect on cell mobility. d-AP5 significantly increased mobility of ECS cells in migration assay (Figure 5G and 5H); in line with this, exogenous administration of NMDA inhibited cell migration (Figure 5I and 5J).

## DISCUSSION

Secreted signaling factors within niches control cancer cells and stem cells [16–18]. As intimate extrinsic factors for the cell regulation, autocrine signaling cues are released and responded to by a same cell type; moreover, they also can act on nearby other cell types, in paracrine manners, when diffused throughout the microenvironment [19]. Most of these factors investigated so far are secreted signaling proteins. Discovering novel autocrine cues such as the endogenous active small- molecule glutamate for cancer stem cell regulation and elucidating its signaling machinery operation will advance our understanding of cancer stem cell biology. This will ultimately help to develop pharmacological strategies targeting the components of the glutamatergic transmission for the control of the cancer stem cells.

VGLUT1 and VGLUT2 are exclusively present in glutamatergic neurons and non-neuronal glutamatergic cells and characterized as markers, sufficing for the definition of the glutamatergic phenotype [20, 21]. ECS cells expressed VGLUT1 and/or VGLUT2, indicating their glutamatergic identification. Combined expression of GLS and VGLUT1/2 reconfirmed the glutamatergic feature of the ECS cells and indicated a glutamatergic signaling output system in these cells.

By using the HPLC-MS/MS approach which ensures superior sensitivity and selectivity for the quantitative analysis of the target small molecule compounds in the complex biological fluid matrix [22, 23], released glutamate from ECS cells was quantitatively determined. The release of glutamate achieved a plateau, implicating an attainment of equilibrium between the release and the termination of the molecule in the extracellular milieu [24, 25], because of the expression of EAATs for reuptake of the released glutamate back into these cells. The equilibration concentrations of the released glutamate in the medium were about 7 μM in ECS cells. These concentrations are sufficient for the activation of the glutamate NMDA receptors [26, 27]. In this study, glutamate at concentrations above 1 μM significantly elicited NMDA receptor activation in ECS cells in terms of the receptor-depended calcium influx.

Genetic knockdown or pharmacological blockade of NMDA receptors augments the proliferation and migration phenotypes of the undifferentiated ECS cells which grew in the exogenous glutamate-free media, further consolidating the autocrine source of glutamate on the regulation of ECS cells. NMDA receptor blockade or knockdown increased the cell DNA synthesis in respect to EdU incorporation and the portion of cells in S cell cycle phase, suggesting that the NMDA receptor activation stimulated by autocrine glutamate is apt to limit ECS cell proliferation through affecting the cell cycle progression. This kind of negative regulatory feedback loop has been proven to control stem cell pool size and cancer growth. Autocrine γ-Aminobutyric acid (GABA), a classic neurotransmitter, lead to the S phase arrest of the cell cycle in ES cells [28, 29].

NMDA receptor activation and coupled calcium influx elicit both positive and negative effects on cell proliferation depending on excitation paradigm and the cell types [30]. Activation of the receptor increases neurogenesis in dentate gyrus and antagonism of the receptor blocks the neuronal progenitor proliferation in the ventricular and the subventricular zone [30–33]. This effect is partly attributed to the mechanisms such as receptor activation-induced Rb phosphorylation, calcium/calmodulin/CREB stimulation, and mitogenic factor release [30, 32]. Other reports show that activation of NMDA receptors decreases cell DNA synthesis and inhibits cell cycle progression whereas blockade of the receptors elicits the opposite effect in hippocampal neural stem/precursors and other cells with the mechanisms involving receptor-coupled modulation of cell cycle machinery and calcium-mediated repression of IGF1R/PI3K/Akt signaling pathway [34–40]. In some human cancers NMDA receptor exhibits tumor-suppressive role and mutations in the receptor have a tumorigenic effect [10, 41, 42]. The suppression of cell outgrowth and migration by NMDA receptors has also been identified in other cell types such as keratinocytes [38].

Therefore, ECS cells express GLS for synthesizing glutamate, VGLUT for loading the glutamate into secretory vesicles, released glutamate for acting as the signal, NMDA receptor for receiving and responding to the released glutamate to supress cell population and mobility, and EAAT for terminating the signal. In this way, a stem cell niche synapse [43, 44] for glutamatergic transmission is formed by ECS cells through an autocrine/paracrine mechanism for the regulation of cell homeostasis. As extrinsic factors, autocrine/paracrine cues precisely control stem cell and cancer stem cell functions by establishing niches and feedback loops [45–49]. Autocrine/paracrine glutamate niche has been detected in pancreatic islet and some kinds of cancers. Pancreatic α cells release glutamate to activate GluRs, which initiates a glucagon secretion, forming a glutamate autocrine feedback loop [5]. In human cancers, interstitial flow elevates the secretion of glutamate, constituting an autocrine signaling circuit via acting on NMDA receptors [8]. In this respect, components in the glutamatergic transmission circuit would represent potential novel targets for interfering ECS cells.

## MATERIALS AND METHODS

### Cell culture and xenograft model

Mouse ECS cell line P19 and human ECS cell line NCCIT were obtained from the Cell Bank of Type Culture Collection of the Chinese Academy of Sciences (Shanghai, China) and the American Type Culture Collection (ATCC), respectively. The cell culture and the pluripotency monitoring were performed as indicated in the previous description [29, 50, 51]. ECS cells were expanded in DMEM (Life Technology, Catalog Number 11995, glutamate free) supplemented with 10% fetal bovine serum (FBS) and 1% GlutaMAX (glutamine). During the assays for the inhibition of endogenous activation of glutamate receptors by the receptor blockers or RNA interference, ECS cells were cultured in serum-free DMEM to ensure the cells were maintained in an exogenous glutamate-free environment. Cells were maintained at 37°C in a humidified atmosphere containing 5% CO_2_ and 95% air.

Transplanted teratocarcinoma was modeled by inoculating mouse ECS cells into the right flank of the athymic BALB/c mice obtained from the the Shanghai Laboratory Animal Center (Chinese Academy of Sciences, Shanghai, China). When tumors of 1-2 cm diameter became established, the mice were euthanized and the tumors were fixed in formalin. The tissues were fixed and sectioned into 4 μm slices for standard hematoxylin and eosin (HE) staining or immunohistochemical/immunofluorescence (IHC/IF) staining. The animal experiment employed in this study was performed in accordance with local ethical guidelines.

### RNA isolation and PCR

Total RNA from cells was isolated with TRIzol reagent (Invitrogen) according to the manufacturer's instructions. Reverse transcription was carried out using a RevertAid First Strand cDNA Synthesis Kit (Qiagen). cDNA was amplified according to the following temperature profile: 94°C for 30 s, 55°C for 45 s, and 72°C for 1 min. At the end of 31 cycles, the reaction was continued for an additional 10 min at 72°C. The amplified products were electrophoresed on 2% agarose gels. The primer sequences used for the reverse transcript-PCR are indicated in Supplementary Table S1A.

### Western blotting and immunohistochemical/immunofluorescence staining

Cell lysates were separated by 12% SDS-PAGE and immunoblotted with the antibodies against Oct4 (1:500, Santa Cruz), VGLUT2 (1:500, Santa Cruz), GLS (1:500, Abcam), or EAAT1 (1:500, Abcam). The membranes were then incubated with corresponding secondary antibodies. The immunoblots were visualized and scanned using an imaging system (Odyssey FC, LI-COR Biosciences).

Immunohistochemical staining was performed on formalin-fixed preparations from human teratocarcinoma tissue slices obtained from the tissue bank of Shanghai Cancer Hospital. The tissue slices were incubated with primary antibodies and subsequently with secondary antibodies. After incubation with VECTASTAIN® ABC Reagent for 30 min, peroxidase activity was developed with DAB Substrate–Chromogen System (Merck). After a final wash in PBS, the slices were counterstained with hematoxylin. Immunofluorescence staining was performed on stem cells and mouse transplanted teratocarcinoma tissue slices. They were incubated with corresponding antibodies. In some cases, dual immunofluorescence staining was performed to investigate whether the transmission components colocalized in the same cells. The primary antibodies used were anti-Oct4 (1:500), anti–GLS (1:100), anti-VGLUT2 (1:100), and anti-EAAT1 (1:500).

### Determination of cytosolic free calcium

After incubated with 1 μM free-calcium probe Fluo-4 AM (Dojindo) at 37°C for 10 min, living cells were scanned at 1 sec intervals using confocal microscopy (Zeiss LSM 510). Increase of fluorescence ratio at the same point indicates an increase in free intracellular Ca^2+^ ([Ca^2+^]i). The analysis of [Ca^2+^]i were processed in a single cell level, and the results were expressed as the fluorescent intensity (F/F0 %, arbitrary unit, F is the fluorescence captured at a particular time and F0 is the initial fluorescence image captured) unless otherwise indicated.

### Measurement of glutamate release using liquid chromatography-tandem mass spectrometry

Calibration standards were prepared at concen- trations of 5, 10, 25, 50, 100, 250 and 500 ng/mL. The stable isotope-labeled glutamate-d5 (C/D/N Isotopes) was used as the internal standard (IS) in the calibration standards. Liquid chromatography was performed using an Agilent 1200 HPLC system (Agilent Technologies), and separation was carried out at 40°C using a ZIC-HILIC column (2.1 mm × 100 mm, 3 μm; Merck). The HPLC system was coupled to an Agilent 6410 triple quadruple mass spectrometer (Agilent Technologies). The mode of MRM was used to identify and quantify glutamate and IS (Supplementary Figure S1, upper panel and down panel, respectively).

The method was validated in terms of selectivity and linearity. For selectivity, blank Krebs-HEPES buffer (KHB) solutions with or without glutamate and IS were analyzed to determine whether there was any interfering compound for glutamate and IS in blank KHB. Calibration curves were constructed using 7 calibration standards for glutamate in the range of 5–500 ng/mL by determining the best-fit of the peak area ratios of analyte to IS (y) vs nominal concentration (x) and fitted to the equation y = bx + a by using 1/x weighted least-squares regression. The linearity of the relationship between the peak area ratio and the concentration was demonstrated by the correlation coefficient (R) obtained with the weighing factor of 1/x. The concentrations of the real samples were calculated according to the equation of the calibration curve.

To determine glutamate release, ECS cells were plated at a density of 3 × 10^6^ cells/well (10 cm diameter). After they attached to the dishes, the cells were washed 3 times with 10 mL KHB to remove endogenous secretions in the old medium and then were incubated in 8 mL of fresh KHB. For the time course experiments, the supernatants, comprising glutamate released by the cells, were removed for analysis at time points of 3, 5, 10, 15, 30 and 60 min after the addition of the fresh KHB. An equal volume of fresh KHB was added back into the corresponding culture dishes after each sampling.

### RNA interference

Glutamate receptor subunit short hairpin RNA (shRNA) oligonucleotides were purchased from Santa Cruz Biotechnology. The RNA interference procedure was performed according to the manufacturer's instruction. Levels of the receptor mRNA were analyzed by real-time quantitative PCR (qPCR). Reactions were carried out in triplicate and repeated twice with a sequence detection system (Lightcycler 480II, Roche). The PCR amplification was performed in 96-well optical reaction plates for 40 cycles, each at 94°C for 20 s and 60°C for 1 min. The primer sequences used are shown in Supplementary Table S1B.

### WST assay

WST-8 colorimetric assay for determination of cell viability was performed using Cell Counting Kit-8 (Dojindo) according to the manufacturer's instruction. The cells were treated with d-AP5, LY341495, or NBQX (all from Tocris Bioscience) at different concentrations for 24 h. Cell viability was assayed by reading the absorbance at 450 nm using a microplate reader (Scientific Vario, Thermo Scientific).

### Flow cytometry

After the cells were exposure to NBQX, D-AP5 and LY341495, they were fixed with 500 μL of 70% ethanol at 4°C for 24 h and incubated with propidium iodide (50 μg/mL) and RNase A (50 μg/mL) at 37°C for 30 min. Samples were analyzed with a flow cytometer (FACS Calibur, Becton Dickinson).

### High-content analysis

Image-based high-content analysis [52, 53] was performed using the HCA System (ArrayScan XTI, Thermo Scientific) as indicated in our previous report [54]. For determination of DNA synthesis, EdU incorporation was detected using Click-iT EdU Imaging Kit (Invitrogen) according to the manufacturer's instruction. Cell nuclei were counterstained with DAPI and the cell pluripotency was monitored by Oct4 immunofluorescence staining. The percentage of proliferating cells was determined by the number of EdU positive nuclei related to the total number of DAPI and/or Oct4 stained nuclei.

### Cell migration assay

Cell migration assays were carried out using transwell chambers (Corning Costar). 1×10^6^ cells suspended in 400 μL of serum-free DMEM were placed in the upper chamber. The lower chamber was filled with 600 μL of DMEM containing different stimulus treatment. After an incubation period of 24 h at 37 °C, the cells on the upper surface of the filter were removed with a cotton swab. Cells adhering to the bottom surface of each membrane were stained with 0.5% crystal violet solution, imaged and counted using a DMR inverted microscope (Leica Microsystems).

### Statistical analysis

Statistical significance was tested using a Student's test or an one-way ANOVA with Bonferroni post-test properly. Differences were considered statistically significant when P<0.05.

## SUPPLEMENTARY MATERIALS FIGURES AND TABLES




