# Supplementary material for: Autocrine glutamatergic transmission for the regulation of embryonal carcinoma stem cells
Source: Oncotarget. 2016 Jun 13;7(31):49552–64. doi: 10.18632/oncotarget.9973 (PMC5226528; doi:10.18632/oncotarget.9973)
Supplement: Supplementary file 1 [file oncotarget-07-49552-s001.pdf]

# Autocrine glutamatergic transmission for the regulation of embryonal carcinoma stem cells

## SUPPLEMENTARY FIGURE AND TABLE

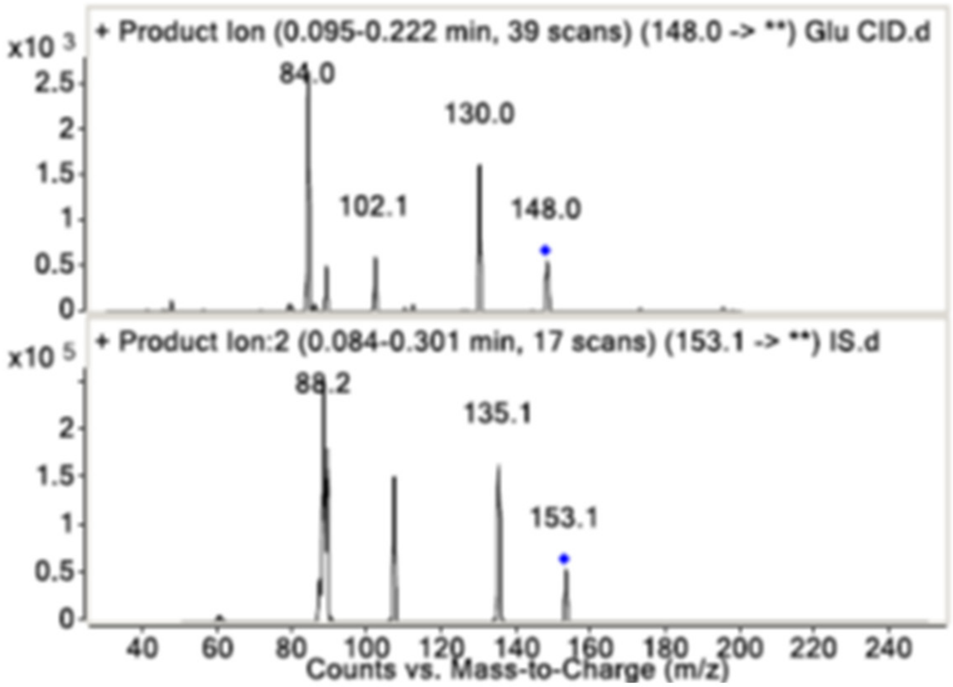


**Supplementary Figure S1: Collision-induced dissociation mass spectra.** Collision-induced dissociation mass spectra of glutamate (upper panel) and IS (down panel). An electrospray ionization (ESI) in positive ionization mode was used. Positive ESI generates mostly protonated ions ($[M + H]^+$) from molecules containing a functional group with the potential for ionization. The positively charged molecular ions m/z 148.0 and 153.1 were generated for glutamate and IS, respectively. Using product ion scan mode, several product ions were generated. Then the most abundant and specific product ion was selected for multiple reaction monitoring (MRM) transition: m/z 148.0 $[M + H]^+ \rightarrow$ 84.0 for glutamate and m/z 153.1 $[M + H]^+ \rightarrow$ 88.1 for IS. IS, internal standard.

**Supplementary Table S1: The primer sequences used for PCR**

**See Supplementary File 1**
