# Supplementary material for: Autocrine glutamatergic transmission for the regulation of embryonal carcinoma stem cells
Source: Oncotarget. 2016 Jun 13;7(31):49552–64. doi: 10.18632/oncotarget.9973 (PMC5226528; doi:10.18632/oncotarget.9973)
Supplement: Supplementary file 2 [file oncotarget-07-49552-s002.doc]

**Supplemental Table 1. The primer sequences used for PCR.**

**(A)** The primer sequences used for RT-PCR

| **Glutamatergic transmission components** | **Gene** | **Primer Sequences (5'-3')** | **Fragment (bp)** |
| --- | --- | --- | --- |
| GluN1 | *Grin1_*F1 | cagaaacccctcagacaagttc | 600 |
|  | *Grin1_*R1 | ggctctgctctaccactctttc |  |
| GluN2A | *Grin2a_*F1 | tcgggtctcatttcagtctctt | 549 |
|  | *Grin2a_*R1 | gcagcacttcttcacattcatc |  |
| GluN2B | *Grin2b_*F1 | tgacttctctgtgcccttcata | 581 |
|  | *Grin2b_*R1 | catcatctacacccctttggtt |  |
| GluN2C | *Grin2c_*F1 | gcactggtcttcaacaactctg | 552 |
|  | *Grin2c_*R1 | tgtctccaacttctgtgtctcc |  |
| GluN2D | *Grin2d_*F1 | aatgaggatggctttctggtaa | 546 |
|  | *Grin2d_*R1 | ctctacaaaagggacggagaag |  |
| GluN3A | *Grin3a_*F1 | ttgtaggggatggaaagtatgg | 592 |
|  | *Grin3a_*R1 | gttccaaaacgaaaaccttgag |  |
| GluN3B | *Grin3b_*F1 | ctatccagttacacagccaacct | 542 |
|  | *Grin3b_*R1 | aggacaaacaaccctgacaagt |  |
|  | *Grm8_*R1 | acgacaaaccacacaaacactc |  |
| Gls | *Gls_*F1 | gtgttggtctcctcctcttgac | 467 |
|  | *Gls_*R1 | catcatcagaatccccttgag |  |
| VGLUT1 | *VGLUT1_*F1 | ctggctatccttctgcacttct | 482 |
|  | *VGLUT1_*R1 | ggtcccattacaaaccctgata |  |
| VGLUT2 | *VGLUT2_*F1 | gcttctggttgttggctactct | 447 |
|  | *VGLUT2_*R1 | gaggtagcaccgtaagatttgg |  |
| VGLUT3 | *VGLUT3*_F1 | ccataccaaaggagtggctatc | 451 |
|  | *VGLUT3_*R1 | ggtcttctggacctcacaattc |  |
| EAAT1 | *EAAT1_*F1 | gtcctgcctctcctctacttcc | 558 |
|  | *EAAT1_*R1 | gttctcctcaatcaccgagttc |  |
| EAAT2 | *EAAT2_*F1 | tttccagcagattcagacagtg | 562 |
|  | *EAAT2_*R1 | gaaggtaacaggcaaagttcca |  |
| EAAT3 | *EAAT3_*F1 | acatcaacaggacgggtaaaac | 538 |
|  | *EAAT3_*R1 | aaatagagcaggggcagaacta |  |
| EAAT4 | *EAAT4_*F1 | cccacatcctcagtagaaaatga | 520 |
|  | *EAAT4_*R1 | ggaaagtgataggcagagttgc |  |
| EAAT5 | *EAAT5_*F1 | tcctgtctgtgctctctgtcat | 445 |
|  | *EAAT5_*R1 | gacttgataactggggtggtct |  |
| β actin | *β actin _*F1 | gagaccttcaacaccccagc | 446 |
|  | *β actin _*R1 | ccacaggattccatacccaa |  |

**(B)** The primer sequences used for real time-qPCR

|  | **Gene** | **Primer Sequences (5'-3')** | **Fragment (bp)** |
| --- | --- | --- | --- |
| GluN2A | *Grin2a_*F1 | ctctgtctgggtgatgatgttc | 223 |
|  | *Grin2a_*R1 | tatcttgctggttgtgccttta |  |
| β actin | *β actin _*F1 | gtccctcaccctcccaaaag | 266 |
|  | *β actin _*R1 | gctgcctcaacacctcaaccc |  |
